# Association of peripheral nerve conduction in diabetic neuropathy with subclinical left ventricular systolic dysfunction

**DOI:** 10.1186/s12933-015-0213-4

**Published:** 2015-05-07

**Authors:** Yasuhide Mochizuki, Hidekazu Tanaka, Kensuke Matsumoto, Hiroyuki Sano, Hiromi Toki, Hiroyuki Shimoura, Junichi Ooka, Takuma Sawa, Yoshiki Motoji, Keiko Ryo, Yushi Hirota, Wataru Ogawa, Ken-ichi Hirata

**Affiliations:** Department of Internal Medicine, Division of Cardiovascular Medicine, Kobe University Graduate School of Medicine, 7-5-2, Kusunoki-cho, Chuo-ku Kobe, 650-0017 Japan; Department of Internal Medicine, Division of Diabetes and Endocrinology, Kobe University Graduate School of Medicine, Kobe, Japan

**Keywords:** Diabetes mellitus, Diabetic neuropathy, Nerve conduction study, F-wave latency, Echocardiography, 2-dimensional speckle-tracking strain, Global longitudinal strain

## Abstract

**Background:**

Subclinical left ventricular (LV) longitudinal myocardial systolic dysfunction occurs in patients with diabetes mellitus (DM) and preserved LV ejection fraction (LVEF), and is closely related to DM-related complications. However, the association of diabetic neuropathy (DN) with subclinical LV systolic longitudinal dysfunction in such patients has not been fully clarified.

**Methods:**

The subjects of this study were 112 consecutive DM patients with preserved LVEF (all ≥50%) without coronary artery disease and overt heart failure (aged 59 ± 14 years; 60 women, 52 men). Global longitudinal strain (GLS) was determined as the average peak strain of 18 segments from the three standard apical views, and was expressed as an absolute value. DN was diagnosed by experienced diabetologists. Median, ulnar, and sural nerves were subjected to motor and sensory nerve conduction studies. F-wave latency was defined as the minimum F-wave latency after a total of 16 stimulations of the tibial nerve.

**Results:**

Forty-one (37%) patients were clinically diagnosed with DN. LV functions of DM patients with and without DN were similar except for GLS being significantly smaller in patients with than in patients without DN (18 ± 2% vs. 20 ± 2%, p < 0.001). It was noteworthy that, of the parameters for the nerve conduction study, only F-wave latency correlated with GLS (r = −0.34, p < 0.001), and also was identified as an independent determinative value of GLS in a multivariate linear regression model (β = −0.25, p = 0.001) even after adjustment for other closely related GLS factors.

**Conclusions:**

Monitoring of F-wave latency may aid early detection of not only DN but also subclinical LV dysfunction. Joint planning of assessment by diabetologists and cardiologists is therefore advisable for better management of DM patients.

## Introduction

Subclinical left ventricular (LV) myocardial dysfunction occurs in patients with diabetes mellitus (DM) and preserved LV ejection fraction (LVEF) but without coronary artery disease or hypertension [1-3]. This condition is considered a major contributor to the development of a type of heart failure (HF) known as diabetic cardiomyopathy. In addition, LV longitudinal myocardial systolic dysfunction may constitute the first marker of a preclinical form of diabetic cardiomyopathy in DM patients with preserved LVEF without overt HF [4]. In particular, global longitudinal strain (GLS) as assessed by two-dimensional speckle-tracking strain is reportedly effective for the assessment of subclinical LV myocardial dysfunction in DM patients with preserved LVEF [4-7]. Many DM-related pathophysiological mechanisms lead to harmful changes including free acid metabolism, increased apoptosis, activation of the renin-angiotensin system or autonomic neuropathy, as well as an increase in oxidative stress or myocardial steatosis, have been found to play an important role in the development of diabetic cardiomyopathy [1,8-10]. Moreover, it has been reported that DM-related complications or patient characteristics such as diabetic nephropathy, diabetic retinopathy, obesity, insulin resistance, or autonomic neuropathy are associated with subclinical LV myocardial dysfunction, because all of them are considered to be accumulative phenotypes of poor metabolic control [11-17]. Severe diabetic neuropathy (DN) also reflects poor blood sugar control, which leads to painful nerve damage as a result of increased deposition of sorbitol, fructose, and advanced glycation end products. Uncontrolled hyperglycemia generates microvascular ischemia, which results in peripheral nerve damage due to vasoconstriction and microvasculopathy [18]. Although this mechanism of DN is the same as that of diabetic cardiomyopathy, the relationship of diabetic peripheral neuropathy with impaired LV longitudinal myocardial systolic function has not yet been fully clarified. Accordingly, the purpose of the study presented here was to determine the relationship between DN and subclinical LV longitudinal myocardial systolic dysfunction in DM patients with preserved LVEF. We also assessed the relationship between the parameters of nerve conduction with LV longitudinal myocardial systolic function in such patients.

## Methods

A total of 118 consecutive DM patients who were admitted to Kobe University Hospital between July 2013 and January 2015 were recruited, and data sets of nerve conduction and echocardiographic studies were prospectively collected. The diagnosis of DM was based on the World Health Organization criteria [19]. Preliminary exclusion criteria for this study were as follows: (1) ischemic heart disease; (2) LVEF < 50%; (3) a previous history of open-heart surgery and congenital heart disease; (4) serious renal dysfunction defined as glomerular filtration rate (GFR) < 30 mL/min/1.73 m^2^; (5) uncontrolled hypertension >180/100 mmHg; (6) significant valvular heart disease; (7) atrial fibrillation; (8) left bundle branch block. Each patient underwent exercise stress testing such as treadmill exercise or stress myocardial perfusion scintigraphy within 2 weeks after admission. The study protocol was approved by the ethics committee of our institution and all patients gave informed consent before participating in this study.

### Echocardiography

All echocardiographic data were obtained by means of a commercially available echocardiographic system (Vivid E9; GE-Vingmed, Horten, Norway) within 2 weeks after admission. Digital routine grayscale two-dimensional cine loops from three consecutive heartbeats were obtained at end-expiratory apnea from the standard parasternal long-axis and three apical views with mean frame rates of 66 ± 7 frames/sec. Sector width was optimized to allow for complete myocardial visualization with maximization of the frame rate. Digital data were transferred to dedicated software (EchoPAC version 113; GE Vingmed) for subsequent offline analysis. Standard LV measurements were obtained in accordance with the current guidelines of the American Society of Echocardiography/European Association of Cardiovascular Imaging [20]. LV volumes and LVEF were calculated using the modified biplane Simpson’s method. Left atrial volume was also calculated with the modified biplane Simpson’s method using apical 2- and 4-chamber views at the ventricular end-systole, and then normalized to body surface area. LV stroke volume was determined in terms of the velocity-time integral assessed by means of pulsed-wave Doppler positioned at the LV outflow tract. The early diastolic (E) and atrial wave velocities and the E-wave deceleration time were measured by using the pulsed-wave Doppler recording from the apical four-chamber view. Spectral pulsed-wave Doppler-derived early diastolic velocity (E’) was obtained from the septal mitral annulus.

### Assessment of GLS

**Figure 1:**
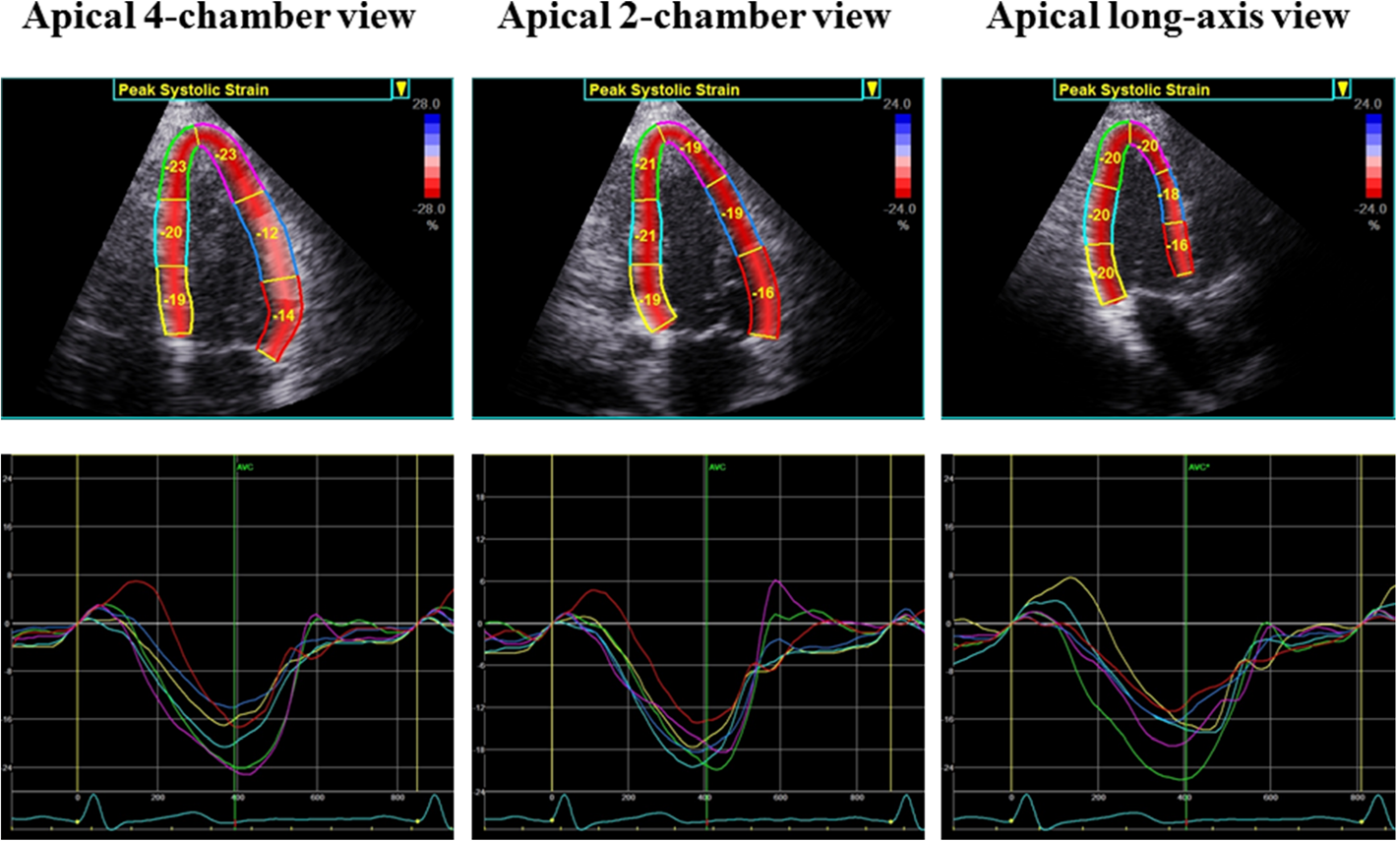
An example of a color-coded two-dimensional display of the left ventricle (LV) and corresponding time-strain curves from 18 LV sites derived from the three standard apical views for measurement of global longitudinal strain (GLS). GLS was determined as the average peak strain of 18 LV segments, and was expressed as an absolute value.

GLS was assessed by means of two-dimensional speckle-tracking longitudinal strain from the three standard apical views with the aid of a single dedicated software (EchoPAC version 113; GE Vingmed) as previously described in detail. Briefly, a region of interest was traced on the endocardium at end-systole with a point-and-click approach for each of the three apical views. A second larger region of interest was then generated and manually adjusted near the epicardium. Each apical image was divided into six standard segments, followed by the generation of six corresponding time-strain curves (Figure 1). GLS was determined as the averaged peak strain of 18 segments from the three standard apical views [20], and was expressed as an absolute value. The physicians who evaluated GLS were blinded to patient data including nerve conduction parameters.

### Nerve conduction study

**Figure 2:**
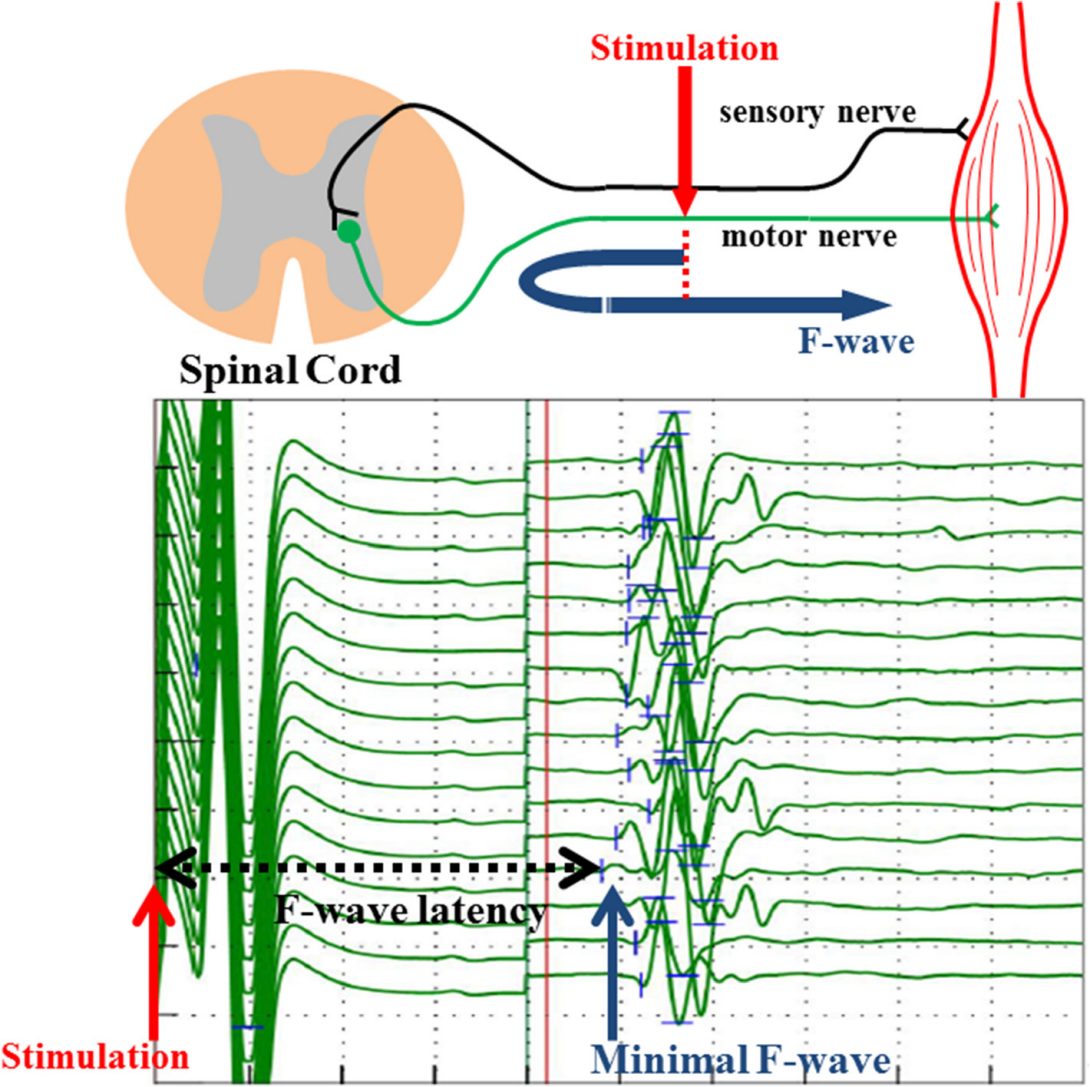
Schema of F-wave combined conduction time for antidromic and orthodromic conduction after 16 stimulations of the tibial nerve, and an actual minimal F-wave latency recording with 16 lines from stimulation to the first occurrence of F- wave.

Sensory and motor nerve conduction velocities for the median, ulnar and sural nerves were obtained from surface recordings and determined by means of conventional techniques [21]. The F-wave is considered to be an electrophysiological artifact bypassing the spinal cord after antidromic activation of motor neurons following distal electrical stimulation of motor nerve fibers. F-wave latency reflects the combined conduction time which consists of the times for antidromic conduction from the site of the stimulation of the peripheral nerve to the spinal cord, and for orthodromic conduction for reactivation of the motor neurons from the spinal cord to the peripheral motor nerve (Figure 2). In this study, F-waves were obtained from the tibial nerve with surface electrodes placed on the on lower leg muscle, and F-wave latency was defined as the minimal F-wave latency in a series of 16 tibial nerve stimulations [22,23]. F-wave occurrence was expressed as a percentage of the F-wave appearance rate for 16 stimulations.

### Collection of baseline clinical data

HemoglobinA1c, glycoalbumin, estimated glomerular filtration rate and lipid profile were obtained the day after admission. Albuminuria was quantitated after 24-hour urine collection. All subjects were interviewed in detail and underwent a thorough neurologic examination which included sensory tests for light touch, pin-prick, and vibration and tendon reflexes. Experienced diabetologists methodically and comprehensively assessed DN according to the current guidelines which combine a patient’s symptoms, neurological findings and results of a nerve conduction study [24]. In addition, diabetic retinopathy was defined as a condition more serious than simple retinopathy or previously treated by laser photocoagulation was based on the evaluation of retinal photographs by experienced ophthalmologists.

### Statistical analysis

Continuous variables were expressed as mean values and their standard deviation for normally distributed data and the median and interquartile range for non-normally distributed data. Categorical variables were expressed as frequencies and percentages. The parameters of the two subgroups were compared by using the Student’s *t*-test or the Mann–Whitney *U* test as appropriate. Proportional differences were evaluated with Fisher’s exact test. Relationships between two variables were analyzed by using linear regression and are expressed as Pearson correlation coefficients. A multiple logistic regression analysis was performed to detect the most sensitive marker of nerve conduction for the prediction of DN using stepwise selection with the p levels for entry from the model set at <0.10.

Since many parameters were associated with GLS, baseline clinical data were first divided into the following four categories to clarify the independency:

1. Demographics and comorbidities: age, gender, body mass index, DM duration, DM type, retinopathy, and dyslipidemia
2. Blood exam and urinary test: HbA1c, glycoalbumin, low-density lipoprotein, triglyceride, estimated glomerular filtration rate and log-transformed albuminuria
3. Cardiac signs: systolic blood pressure, heart rate and rate pressure product
4. All parameters in nerve conduction study: motor and sensory nerve conduction velocities obtained for the median, ulner, and sural nerves, F-wave latency and F-wave occurrence

Second, we chose determinative values for GLS by means of stepwise multivariate regression analysis for each group, with a cut-off p value of 0.15 selected so as not to miss potentially influential parameters. We then constructed the final multivariate regression model consisting of the variables selected from four models. The intraclass correlation coefficient was used to determine inter- and intra-observer reproducibilities for GLS from 15 randomly selected patients using identical cine-loops. Statistical significance was set at p < 0.05. All analyses were performed with SPSS version 16.0 (SPSS, Inc., Chicago, IL).

## Results

### Baseline characteristics

Six patients (5%) were excluded from all analyses subsequent to echocardiographic examinations because of suboptimal images from poor quality echocardiographic windows. As a result, the final study population consisted of 112 patients (Table 1). Their mean age was 59 ± 14 years, LVEF was 66 ± 4%, and 60 patients (54%) were female. Eighty-seven patients (78%) were diagnosed as type 2 DM, and the remaining 25 patients (22%) were classified as Type 1 DM. The intraclass correlation coefficients for intra- and inter-observer reproducibility for GLS were 0.95 (95% confidence interval (CI): 0.855- 0.982) and 0.92 (95% CI: 0.775-0.973), respectively.

**Table 1:** **Clinical, Hemodynamic, and Echocardiographic Characteristics of Patients**

	**All Patients (n = 112)**	**Patients with DN (n = 41)**	**Patients without DN (n = 71)**	**p value**
***Clinical Data***				
Age, years	59 ± 14	61 ± 15	59 ± 12	0.40
Female, n (%)	60(54)	17(41)	43(61)	0.08
Height, m	1.60 ± 0.96	1.62 ± 0.10	1.59 ± 0.09	0.06
Weight, kg	63 ± 15	66 ± 18	61 ± 18	0.12
Body Mass Index, kg/m^2^	24 ± 5	25 ± 6	24 ± 4	0.48
Systolic blood pressure, mmHg	125 ± 18	126 ± 16	124 ± 18	0.51
Heart rate, bpm	73 ± 11	75 ± 11	72 ± 10	0.09
Rate Pressure Product, bpm*mmHg	9132 ± 1938	9501 ± 1770	8925 ± 1997	0.12
Type 2 DM, n (%)	87(78)	33(80)	54(76)	0.64
DM duration, years	11 ± 9	15 ± 9	9 ± 8	0.001
Hypertension, n (%)	55(49)	20(49)	35(49)	1.00
Dyslipidemia, n (%)	71(63)	28(68)	43(61)	0.54
Smoking, n (%)	24(21)	8(20)	16(23)	0.81
Retinopathy, n (%)	42(38)	22(54)	20(28)	0.009
***Biochemistry and Urinary Exam***				
HbA1c, %	8.6 ± 2.2	8.7 ± 2.4	8.6 ± 2.1	0.75
Glycoalbumin, %	23.9 ± 9.1	24.9 ± 10.5	23.4 ± 8.1	0.42
Low-density lipoprotein, mg/dl	105 ± 35	101 ± 37	107 ± 34	0.41
Triglyceride, mg/dl	135 ± 73	130 ± 65	138 ± 77	0.55
eGFR, ml/min/1.73 m^2^	74 ± 23	68 ± 23	77 ± 22	0.048
Albuminuria, mg/day	10(4–31)	15(6–117)	7(3–21)	0.007
***Medical treatment***				
CCB, n (%)	27(24)	7(17)	20(28)	0.25
ACEI/ARB, n (%)	45(40)	17(41)	28(39)	0.83
β-blocker, n (%)	8(7)	2(5)	6(8)	0.71
Statin, n (%)	51(46)	24(59)	27(38)	0.049
Insulin, n (%)	65(58)	26(63)	39(55)	0.43
DPP-4I, n (%)	46(41)	20(49)	26(37)	0.24
GLP-1RA, n (%)	12(11)	5(12)	7(10)	0.76
Sulfonylurea, n (%)	23(21)	8(20)	15(21)	1.00
αGI, n (%)	22(20)	11(27)	11(15)	0.22
Thiazolidine, n (%)	13(12)	7(17)	6(8)	0.22
Metformin, n (%)	50(45)	20(49)	30(42)	0.56

Values are mean ± SD for normally distributed data and median and interquartile range for non-normally distributed data, or n (%).

DN = diabetic neuropathy; DM = diabetes mellitus; eGFR = estimated glomerular filtration rate; CCB = calcium channel blocker; ACEI = angiotensin-converting enzyme inhibitor; ARB = angiotensin II receptor blocker; DPP-4I = dipeptidyl peptidase-4 inhibitor; GLP-1RA = glucagon like peptide-1receptor agonist; α-GI = α-glucosidase inhibitor.

### Baseline clinical data in DM patients with and without DN

Forty-one (37%) patients were clinically diagnosed with DN by experienced diabetologists. Comparison of baseline clinical characteristics of patients with and without DN is shown in Table 1. Patients with DN had significantly longer DM duration (15 ± 9 years vs. 9 ± 8 years, p = 0.001) and higher prevalence of retinopathy (54% vs. 28%, p = 0.009) and use of statin (59% vs. 38%, p = 0.049) compared to those without DN. Albuminuria (15 [6–117] mg/day vs. 7 [3-21] mg/day, p = 0.007) was significantly larger for patients with than without DN. Parameters indicating the extent of recent blood sugar control and the parameters of afterload were similar for the two groups.

### Nerve conduction study

Comparison of nerve conduction parameters and the results of a multiple logistic regression analysis for DN detection are given in Table 2. As expected, patients with DN showed significant impairment in almost all parameters examined in the nerve conduction study. Motor nerve conduction velocities for the median and sural sensory nerves of patients with DN were significantly slower for those of patients without DN (51 ± 5mesc vs. 53 ± 4mesc, p = 0.02; 41 ± 4mesc vs. 44 ± 5mesc, p < 0.001, respectively). Similarly, sensory nerve conduction velocities for the median, ulner, and sural sensory nerves were significantly slower for patients with than without DN (45 ± 6mesc vs. 50 ± 7mesc, p < 0.001; 46 ± 5mesc vs. 50 ± 6mesc p < 0.001; 46 ± 6mesc vs. 49 ± 7mesc, p = 0.04, respectively). F-waves during 16 stimulations occurred significantly less often in patients with DN than in those without DN (87% vs. 96%, p = 0.04). Furthermore, significantly longer F-wave latency was observed in patients with DN than in those without DN (53 ± 6 vs. 48 ± 5, p < 0.001). An important finding of multiple logistic regression was that F-wave latency was the strongest predictor for DN independently from other parameters (odds ratio = 1.18, 95%CI: 1.062-1.319; p = 0.002).

**Table 2:** **Nerve Conduction Study of the Patients**

	**Comparison in both groups**			**Multivariate logistic regression for DN**		
	**Patients with DN (n = 41)**	**Patients without DN (n = 71)**	**p value**	**OR**	**95% CI**	**p value**
***Motor nerve conduction velocities***						
median nerve, msec	51 ± 5	53 ± 4	0.02			
ulnar nerve, msec	51 ± 5	52 ± 5	0.32			
sural nerve, msec	41 ± 4	44 ± 5	<0.001			
***Sensory nerve conduction velocities***						
median nerve, msec	45 ± 6	50 ± 7	<0.001	0.92	0.851 - 0.993	0.03
ulnar nerve, msec	46 ± 5	50 ± 6	<0.001			
sural nerve, msec	46 ± 6	49 ± 7	0.04			
***F-wave( tibial nerve)***						
F-wave occurrence, %	87	96	0.04			
F-wave latency, msec	53 ± 6	48 ± 5	<0.001	1.18	1.062 - 1.319	0.002

Values are mean ± SD.

DN = diabetic neuropathy; OR = odds ration; 95% CI = 95% confidential interval.

### Comparison of echocardiographic parameters for patients with and without DN

Results of a comparison of echocardiographic parameters for patients with and without DN are summarized in Table 3. The standard echocardiographic parameters for the two groups were similar except for GLS for patients with DN being significantly smaller than for patients without DN (18 ± 2% vs. 20 ± 2%, p < 0.001).

**Table 3:** **Echocardiographic Characteristics of the Patients**

	**All Patients (n = 112)**	**Patients with DN (n = 41)**	**Patients without DN (n = 71)**	**p value**
Left atrial volume index, ml/m^2^	29 ± 8	31 ± 9	28 ± 7	0.18
LV mass index, g/m^2^	76 ± 18	79 ± 18	74 ± 18	0.17
End systolic volume, ml	24 ± 9	25 ± 10	24 ± 8	0.42
End diastolic volume, ml	71 ± 19	73 ± 20	70 ± 18	0.37
LV ejection fraction, %	66 ± 4	66 ± 3	66 ± 4	0.96
Stroke volume, ml	63 ± 12	62 ± 14	63 ± 11	0.67
E/A	0.88 ± 0.29	0.82 ± 0.28	0.9 ± 0.29	0.15
Deceleration Time	205 ± 53	203 ± 63	206 ± 46	0.76
E’	6.2 ± 1.8	5.9 ± 1.7	6.4 ± 1.9	0.20
E/E’	10.5 ± 3.3	11.0 ± 3.8	10.2 ± 3.0	0.27
Global longitudinal strain, %	19 ± 3	18 ± 2	20 ± 2	<0.001

Values are mean ± SD for normally distributed data.

DN = diabetic neuropathy; LV = left ventricular; E = peak early diastolic mitral flow velocity; A = peak late diastolic mitral flow velocity; E’ = Spectral pulsed-wave Doppler–derived early diastolic velocity from the septal mitral annulus.

### Relationship between F-wave latency and GLS

We constructed a two-step multivariate analysis for evaluation of independent associations with GLS. Based on the results of the first multivariate linear regression analysis of four pre-defined models, ten individual parameters comprising gender, DM duration, type 2 DM, retinopathy, body mass index, rate pressure product, triglyceride, glycoalbumin, albuminuria and F-wave latency were chosen for the final multivariate linear regression analysis (Table 4). A significant finding was that F-wave latency was one of the independent determinative factors of GLS (β = −0.25, p = 0.001).

**Table 4:** **Univariate and multivariate regression analysis for detecting GLS**

	**Univariate**		**Multivariate**	
**Dependent Variables**	**β**	**p value**	**β**	**p value**
Gender (female)	0.14	0.11		
DM duration, years	−0.16	0.07	−0.18	0.016
Type 2 DM	−0.24	0.007		
Retinopathy	−0.18	0.047		
Body mass index, kg/m^2^	−0.34	<0.001	−0.23	0.003
Rate Pressure Product, bpm*mmHg	−0.40	<0.001	−0.20	0.016
Triglyceride, mg/dl	−0.28	0.001	−0.22	0.006
Glycoalbumin, %	0.15	0.07		
Albuminuria (Log)	−0.43	<0.001	−0.26	0.001
F-wave latency, msec	−0.27	0.006	−0.25	0.001

GLS = global longitudinal strain; DM = diabetes mellitus.

## Discussion

The study presented here provides the first evidence that LV longitudinal myocardial systolic function in asymptomatic DM patients with preserved LVEF is significantly impaired in DM patients with DN compared to those without DN in spite of similar conventional echocardiographic parameters. Furthermore, only F-wave latency, which is considered to be the most sensitive marker of diabetic peripheral neuropathy, was found to be independently associated with subclinical LV longitudinal myocardial systolic function in a nerve conduction study.

### Impact of GLS on diabetic neuropathy

Painful symptoms of diabetic peripheral neuropathy are thought to be triggered by increased depositions of sorbitol, fructose, advanced glycation end products, and free oxygen radicals which reflect uncontrolled hyperglycemia and its sustained duration. In addition, hyperglycemia conveys microvascular ischemia to the peripheral nerves via vasoconstriction and thickening of the basement membrane in blood vessels, resulting in painful damage to the peripheral nerves [18]. Since these pathological mechanisms of DN indicate the degree of diabetic seriousness and the exact duration for DM patients exposed to uncontrolled hyperglycemia [25], the presence of DN can reflect the extent to which LV myocardium has been impaired. Nonetheless, it is not easy to evaluate correctly such often subtle LV myocardial degeneration. Marangoni et al. used streptozotocin-induced rat model of diabetes to prove increased LV volume and impaired systolic and diastolic function just between 4 and 12 weeks after DM onset in parallel with development of insensate neuropathy [26]. Sacre et al. provided evidence of the impaired subclinical LV myocardial systolic and diastolic functions in type 2-DM patients with cardiac autonomic neuropathy [27]. In their study population, 16 (14%) subjects had cardiac autonomic neuropathy, and their peak systolic and early diastolic velocities determined with color tissue Doppler were significantly lower than those of patients without cardiac autonomic neuropathy. This finding suggests that LV longitudinal systolic and diastolic functions are impaired despite preserved LVEF in patients with cardiac autonomic neuropathy.

### F-wave latency and GLS

Nerve conduction studies are generally sensitive, reliable, noninvasive, and objective means of examining diabetic neuropathy. Notably, minimal F-wave latency is a well-established component of nerve conduction studies and is considered to be the most sensitive and reliable of all relevant parameters [28]. In our subjects, a multiple logistic regression model identified F-wave latency as the most powerful predictor for DN. In addition, F-wave latency showed an independent correlation with GLS, suggesting the first evidence verifying the presence of subclinical LV myocardial systolic dysfunction. Previous investigators have demonstrated that activation of dipeptidyl peptidase-4, retinopathy, albuminuria, obesity and hypertriglyceridemia are associated with subclinical LV myocardial dysfunction [11,14,16,29,30]. In fact, almost of these clinical features were evident in the final multiple linear regression model used for the detection of GLS in our population. However, F-wave latency still proved to be a powerful independent determinative factor in addition to albuminuria. Our finding suggests that, among the many potential confounders, subclinical LV myocardial systolic dysfunction is independently associated with DN.

### Clinical implications

The LV wall is not homogenous and is composed of three layers of fibers [31]. LV longitudinal myocardial function is governed by the subendocardial/subepicardial myocardial fibers which are aligned longitudinally so that selective impairment of longitudinal myocardial function may be related to an increase in subendocardial/subepicardial stress. Previous investigators have observed early manifestations of LV abnormalities caused by various diseases including DM in LV longitudinal myocardial function in spite of preserved LVEF [32-34]. Among the various parameters for the assessment of LV longitudinal myocardial function, GLS is thought to be the most reliable marker and to have good reproducibility [35]. Since speckle-tracking strain analysis requires experience to achieve reproducible results and its effective use requires training, it could be difficult to use routinely for general physicians such as diabetologists or even cardiologists who were unfamiliar with speckle tracking. Because LV longitudinal myocardial dysfunction can eventually lead to impaired global LV performance, and the presence of DM may cause changes in LV performance over time, watchful observation of DM patients with preserved LVEF but with impaired F-wave latency by both diabetologists and cardiologists may well prove to be of vital importance. Joint planning of assessment by diabetologists and cardiologists is therefore advisable for better management of DM patients.

### Study limitations

This study covered a relatively small number of patients in a single center study, so that future studies of larger patient populations are needed to verify our findings. Moreover, our study population did not include normal healthy subjects so that the comparison with GLS in DM patients without DN and normal healthy subjects was not part of this study. Finally, 92 patients (82%) underwent treadmill exercise, and the remaining 20 patients (18%) did stress myocardial perfusion scintigraphy to exclude coronary disease in this study. The group who underwent treadmill exercise may potentially include patients with undiagnosed coronary artery disease.

## Conclusions

Monitoring of F-wave latency may aid early detection of not only DN but also subclinical LV dysfunction in asymptomatic DM patients.

## Abbreviations

BMI: Body mass index
CI: Confidence interval
DM: Diabetes mellitus
DN: Diabetic neuropathy
E: Early diastolic wave velocity
E: Spectral pulsed-wave Doppler-derived early diastolic velocity from the septal mitral annulus
EF: Ejection fraction
GFR: Glomerular filtration rate
GLS: Global longitudinal strain
HF: Heart failure

## References

[1] Bando YK, Murohara T (2014). Diabetes-related heart failure. Circ J.

[2] Ryden L, Grant PJ, Anker SD, Berne C, Cosentino F, Danchin N (2013). Esc guidelines on diabetes, pre-diabetes, and cardiovascular diseases developed in collaboration with the easd: the task force on diabetes, pre-diabetes, and cardiovascular diseases of the european society of cardiology (esc) and developed in collaboration with the european association for the study of diabetes (easd). Eur Heart J.

[3] Yancy CW, Jessup M, Bozkurt B, Butler J, Casey DE, Drazner MH (2013). 2013 ACCF/AHA guideline for the management of heart failure: a report of the american college of cardiology foundation/american heart association task force on practice guidelines. J Am Coll Cardiol.

[4] Ernande L, Bergerot C, Rietzschel ER, De Buyzere ML, Thibault H, Pignonblanc PG (2011). Diastolic dysfunction in patients with type 2 diabetes mellitus: is it really the first marker of diabetic cardiomyopathy?. J Am Soc Echocardiogr.

[5] Ernande L, Rietzschel ER, Bergerot C, De Buyzere ML, Schnell F, Groisne L (2010). Impaired myocardial radial function in asymptomatic patients with type 2 diabetes mellitus: a speckle-tracking imaging study. J Am Soc Echocardiogr.

[6] Nakai H, Takeuchi M, Nishikage T, Lang RM, Otsuji Y (2009). Subclinical left ventricular dysfunction in asymptomatic diabetic patients assessed by two-dimensional speckle tracking echocardiography: correlation with diabetic duration. Eur J Echocardiogr.

[7] Ernande L, Bergerot C, Girerd N, Thibault H, Davidsen ES, Gautier Pignon-Blanc P (2014). Longitudinal myocardial strain alteration is associated with left ventricular remodeling in asymptomatic patients with type 2 diabetes mellitus. J Am Soc Echocardiogr.

[8] Maisch B, Alter P, Pankuweit S (2011). Diabetic cardiomyopathy–fact or fiction?. Herz.

[9] Zarich SW, Nesto RW (1989). Diabetic cardiomyopathy. Am Heart J.

[10] Soltysinska E, Thiele S, Olesen SP, Osadchii OE (2011). Chronic sympathetic activation promotes downregulation of beta-adrenoceptor-mediated effects in the guinea pig heart independently of structural remodeling and systolic dysfunction. Pflugers Arch.

[11] Rijzewijk LJ, van der Meer RW, Smit JW, Diamant M, Bax JJ, Hammer S (2008). Myocardial steatosis is an independent predictor of diastolic dysfunction in type 2 diabetes mellitus. J Am Coll Cardiol.

[12] Schuster I, Vinet A, Karpoff L, Startun A, Jourdan N, Dauzat M (2012). Diastolic dysfunction and intraventricular dyssynchrony are restored by low intensity exercise training in obese men. Obesity (Silver Spring, Md).

[13] Karagoz A, Bezgin T, Kutluturk I, Kulahcioglu S, Tanboga IH, Guler A, et al. Subclinical left ventricular systolic dysfunction in diabetic patients and its association with retinopathy: a 2D speckle tracking echocardiography study. Herz. 2014; in press. 10.1007/s00059-014-4138-6 25205476

[14] Takeda Y, Sakata Y, Mano T, Ohtani T, Tamaki S, Omori Y (2011). Diabetic retinopathy is associated with impaired left ventricular relaxation. J Card Fail.

[15] Liu JE, Robbins DC, Palmieri V, Bella JN, Roman MJ, Fabsitz R (2003). Association of albuminuria with systolic and diastolic left ventricular dysfunction in type 2 diabetes: the strong heart study. J Am Coll Cardiol.

[16] Orem C, Kucukosmanoglu M, Hacihasanoglu A, Yilmaz R, Kasap H, Erdogan T (2004). Association of doppler-derived myocardial performance index with albuminuria in patients with diabetes. J Am Soc Echocardiogr.

[17] Utsunomiya H, Yamamoto H, Kunita E, Hidaka T, Kihara Y (2014). Insulin resistance and subclinical abnormalities of global and regional left ventricular function in patients with aortic valve sclerosis. Cardiovasc Diabetol.

[18] Kaur S, Pandhi P, Dutta PD (2011). Painful diabetic neuropathy: an update. Ann Neurosci.

[19] Alberti KG, Zimmet PZ (1998). Definition, diagnosis and classification of diabetes mellitus and its complications. part 1: diagnosis and classification of diabetes mellitus provisional report of a who consultation. Diabet Med.

[20] Lang RM, Badano LP, Mor-Avi V, Afilalo J, Armstrong A, Ernande L (2015). Recommendations for cardiac chamber quantification by echocardiography in adults: an update from the American Society of Echocardiography and the European Association of Cardiovascular Imaging. J Am Soc Echocardiogr.

[21] Pan H, Lin J, Chen N, Jian F, Zhang Z, Ding Z (2013). Normative data of f-wave measures in china. Clin Neurophysiol.

[22] Fisher MA (2007). F-waves–physiology and clinical uses. ScientificWorldJournal.

[23] Antidromic Pan H, Jian F, Lin J, Chen N, Zhang C, Zhang Z (2014). F-wave latencies in patients with diabetes mellitus. Muscle Nerve.

[24] Tesfaye S, Boulton AJ, Dyck PJ, Freeman R, Horowitz M, Kempler P (2010). Diabetic neuropathies: update on definitions, diagnostic criteria, estimation of severity, and treatments. Diabetes Care.

[25] Dyck PJ, Davies JL, Wilson DM, Service FJ, Melton LJ, O'Brien PC (1999). Risk factors for severity of diabetic polyneuropathy: intensive longitudinal assessment of the rochester diabetic neuropathy study cohort. Diabetes Care.

[26] Marangoni MN, Brady ST, Chowdhury SA, Piano MR (2014). The co-occurrence of myocardial dysfunction and peripheral insensate neuropathy in a streptozotocin-induced rat model of diabetes. Cardiovasc Diabetol.

[27] Sacre JW, Franjic B, Jellis CL, Jenkins C, Coombes JS, Marwick TH (2010). Association of cardiac autonomic neuropathy with subclinical myocardial dysfunction in type 2 diabetes. J Am Coll Cardiol Img.

[28] Andersen H, Stalberg E, Falck B (1997). F-wave latency, the most sensitive nerve conduction parameter in patients with diabetes mellitus. Muscle Nerve.

[29] Crendal E, Walther G, Vinet A, Dutheil F, Naughton G, Lesourd B (2013). Myocardial deformation and twist mechanics in adults with metabolic syndrome: impact of cumulative metabolic burden. Obesity (Silver Spring, Md).

[30] Ravassa S, Barba J, Coma-Canella I, Huerta A, Lopez B, Gonzalez A (2013). The activity of circulating dipeptidyl peptidase-4 is associated with subclinical left ventricular dysfunction in patients with type 2 diabetes mellitus. Cardiovasc Diabetol.

[31] Greenbaum RA, Ho SY, Gibson DG, Becker AE, Anderson RH (1981). Left ventricular fibre architecture in man. Br Heart J.

[32] Ng AC, Delgado V, Bertini M, van der Meer RW, Rijzewijk LJ, Shanks M (2009). Findings from left ventricular strain and strain rate imaging in asymptomatic patients with type 2 diabetes mellitus. Am J Cardiol.

[33] Sawaya H, Sebag IA, Plana JC, Januzzi JL, Ky B, Cohen V (2011). Early detection and prediction of cardiotoxicity in chemotherapy-treated patients. Am J Cardiol.

[34] Kato TS, Noda A, Izawa H, Yamada A, Obata K, Nagata K (2004). Discrimination of nonobstructive hypertrophic cardiomyopathy from hypertensive left ventricular hypertrophy on the basis of strain rate imaging by tissue doppler ultrasonography. Circulation.

[35] Barbier P, Mirea O, Cefalu C, Maltagliati A, Savioli G, Guglielmo M. Reliability and feasibility of longitudinal AFI global and segmental strain compared with 2D left ventricular volumes and ejection fraction: intra- and inter-operator, test-retest, and inter-cycle reproducibility. Eur Heart J Cardiovasc Imaging. 2015; in press. 10.1093/ehjci/jeu274 25564395

